# Association between short-term ambient temperature variability (TV, DTR, and TCN) and asthma-related healthcare visit risk in children and adolescents: a systematic review and meta-analysis

**DOI:** 10.3389/fpubh.2026.1902965

**Published:** 2026-08-13

**Authors:** Chengyao Nong, Ping Huang

**Affiliations:** Department of Health Management, The First Affiliated Hospital of Guangxi Medical University, Nanning, Guangxi, China

**Keywords:** childhood asthma, climate change, diurnal temperature range, meta-analysis, temperature variability

## Abstract

**Objective:**

The aim of this study is to systematically evaluate the association between short-term ambient temperature changes (including diurnal temperature range [DTR], temperature change between neighboring days [TCN], and temperature variability [TV]) and asthma-related healthcare utilization among children and adolescents, and to provide evidence for childhood asthma prevention and control under climate change.

**Methods:**

We systematically searched PubMed, Web of Science, Embase, Cochrane Library, and the China National Knowledge Infrastructure (CNKI) database for eligible ecological time-series and case-crossover studies up to 27 May 2026. Risk of bias was assessed using the modified Risk Of Bias In Non-randomized Studies of Interventions (ROBINS-I) tool. Meta-analysis was performed using R software. All effect estimates were unified as relative risks (RR) with 95% confidence intervals (CIs) and pooled using a random-effects model. Subgroup and sensitivity analyses were conducted.

**Results:**

A total of 14 studies were included. Meta-analysis showed that extreme DTR (P_95_/P_99_) was significantly associated with an increased risk of childhood asthma-related visits (RR = 1.72, 95% CI: 1.10–2.71), whereas the linear effect of a 1 °C increase in DTR was not significant (RR = 1.06, 95% CI: 0.99–1.15). Subgroup analysis indicated a stronger effect of DTR in the cold season (RR = 1.18, 95% CI: 1.03–1.35) than in the warm season (RR = 1.05, 95% CI: 0.98–1.12), with no significant interaction by season (*p* = 0.13). No significant sex difference was observed (boys: RR = 1.03, 95% CI: 1.02–1.04; girls: RR = 1.02, 95% CI: 1.00–1.04; *p* = 0.14). Meta-analysis was not performed for TCN and TV_0−t_ because of limited studies and inconsistent exposure definitions, whereas descriptive analysis suggested potential seasonal modifications. Sensitivity analyses indicated that the primary results were stable.

**Conclusion:**

Current quantitative evidence mainly supports an association between extreme DTR and an increased risk of pediatric asthma-related visits. No statistically significant effect modification by season, sex, or age was identified for DTR, although numerical trends were observed. For other temperature variability indicators (TCN and TV_0-t_), definitive conclusions cannot be drawn because of limited available studies and inconsistent exposure definitions. More standardized research with uniform indicator definitions is needed to clarify the independent effects of TCN and TV_0−t_.

**Systematic review registration:**

PROSPERO. Unique Identifier: CRD420261406886; Public registration URL: https://www.crd.york.ac.uk/prospero/display_record.php?RecordID=420261406886.

## 1 Introduction

Asthma is the most prevalent chronic respiratory disease among children worldwide. Despite advances in medical interventions over the past decades, its prevalence continues to increase in many low- and middle-income regions and rapidly urbanizing areas (1, 2). Simultaneously, global warming is altering the environment at an unprecedented rate. Reports from the Intergovernmental Panel on Climate Change (IPCC) confirm that, alongside rising average temperatures, extreme weather events have become more frequent, intense, and prolonged (3). Such climate instability not only poses direct risks to human health but also exacerbates the disease burden of allergic conditions, including asthma (4).

The majority of prior studies have examined the associations between long-term exposure to air pollutants and bioaerosols, such as pollen and mold, and asthma (5, 6). Weather factors, especially temperature fluctuations, are now recognized as major non-pharmacological triggers for acute asthma exacerbations (7). Children have immature thermoregulatory systems, higher breathing rates relative to their body weight, and greater outdoor activities. Their respiratory systems are therefore highly vulnerable to minor changes in atmospheric conditions (8).

Current epidemiological evidence on temperature and asthma mainly focuses on the effects of absolute temperature. It explores associations between extreme heat or cold and disease incidence (9). Multiple studies have shown that heat waves increase the risk of asthma-related emergency department visits (10). Cold surges are strongly linked to sharp increases in pediatric asthma outpatient visits (11). Recent studies indicate that temperature variability (TV) poses a more subtle and widespread risk for people with asthma than extreme individual temperatures. Climate warming alters atmospheric circulation patterns. This leads to a larger diurnal temperature range (DTR) and more frequent sharp temperature fluctuations between consecutive days (TCN) (12). Several studies have analyzed the relationships between DTR or TCN and asthma. However, these studies remain fragmented. Tong et al. (13) conducted research in Shanghai, China. Their results revealed that a higher DTR significantly elevated the risk of pediatric asthma outpatient visits. Jang et al. (14) reported associations between DTR and acute upper respiratory tract infections in Seoul, South Korea. However, these studies were limited to specific geographic regions. Moreover, systematic comparisons across different temperature variability indicators are lacking.

This study conducted a systematic review and meta-analysis that synthesized global epidemiological evidence on short-term ambient temperature variations. The analysis covered DTR, TCN, and other temperature variability indicators, as well as their associations with asthma-related healthcare visit risks among children and adolescents. This study aims to inform evidence-based public health strategies for childhood asthma prevention and control amid ongoing climate change.

## 2 Materials and methods

### 2.1 Study registration

This review strictly followed the PRISMA guidelines. PRISMA refers to Preferred Reporting Items for Systematic Reviews and Meta-Analyses (15). The study protocol was registered in the International Prospective Register of Systematic Reviews (PROSPERO, Registration No. CRD420261406886).

### 2.2 Retrieval strategy

We systematically searched PubMed, Web of Science, Embase, Cochrane Library, and CNKI databases. The search period ranged from the inception of each database to 27 May 2026. To maximize retrieval sensitivity, the search strategy included all studies of short-term temperature fluctuations and asthma. The search terms combined Medical Subject Headings (MeSH) and free-text keywords. Three core concepts were used: ① Exposure factors: “diurnal temperature range,” “temperature variability,” “temperature variation,” and their abbreviations (e.g., DTR, TCN, and TV). ② Outcome measures: “asthma,” “wheeze,” and related respiratory disease terms. ③ Population: No strict age limits were imposed during the initial search. This ensured that studies with pediatric subgroup data were not missed. All database-specific search syntaxes are listed in Supplementary Table S1 to ensure full reproducibility. All search strategies were independently developed by two reviewers and cross-checked for consistency.

### 2.3 Eligibility criteria

We established the inclusion criteria following the population, intervention, comparator, outcomes, and study design (PICOS) framework.

① Study design: Population-based ecological time-series studies or case-crossover studies. These two designs are widely applied observational designs for assessing associations between short-term environmental exposures and acute health outcomes. Case-crossover designs can help adjust for time-invariant individual-level confounding, whereas ecological time-series studies have inherent limitations in controlling for individual-level confounding factors. ② Study population: Children and adolescents aged 0–18 years. Studies covering both adults and children were included only if separate data for pediatric subgroups could be extracted. ③ Exposure factors: Studies must report at least one indicator of short-term ambient temperature changes. DTR: Difference between daily maximum and minimum temperature. TCN: Difference between the mean temperature of the current day and the previous day. TV_0-t_: Standard deviation of daily maximum and minimum temperatures over t consecutive days. The lag periods and cumulative exposure windows must also be clearly stated. For meta-analysis pooling, we unified the analytical reference benchmarks to facilitate cross-study comparisons. These standardized cut-offs (0 °C for linear 1 °C DTR increments, below the 50th percentile for extreme DTR, zero temperature change for TCN and TV_0−t_) were only applied for post-hoc data harmonization and do not represent the native reference groups reported in individual primary studies. Full details of the original study-specific exposure contrasts and reference definitions are presented in Table 1. ④ Outcome measures: Asthma-related healthcare visits, including outpatient visits, emergency department visits, and hospital admissions. ⑤ Quantitative outcomes: Reported extractable effect sizes, such as relative risk (RR) and odds ratio (OR), with 95% confidence intervals (95% CI). Sufficient raw data for calculating these indicators were also accepted. ⑥ Publication type: Peer-reviewed journal articles written in English or Chinese.

**Table 1:** Exposure analysis of included studies.

Study (author, year)	Model	Exposure	Definition	Lag	Lag type	Measure	Unit/contrast	Met. adj.	Pol. adj.
Hu et al., 2020 (19)	Poisson GLM + DLNM	DTR	T_max_–T_min_	lag 0–28 (cold); lag 0–5 (warm)	Cumulative	RR	P_95_ vs. P_50_	Yes	Yes
Hu et al., 2022 (20)	Quasi-Poisson GLM + DLNM	DTR, TCN	DTR: T_max_–T_min_; TCN: T_mean_t_–T_mean_t–1_	lag 0–14	Cumulative	RR	DTR: P_99_ vs. min TCN: P_99_ vs. 0 (no change)	Yes	Yes
Wang et al., 2018 (21)	Poisson GAM	DTR	T_max_–T_min_	lag 0	Single-day/threshold-based	RR	Per 1 °C above threshold (8.8°C)	Yes	No
Lei et al., 2020 (22)	Quasi-Poisson + DLNM	TCN	T_today_–T_yesterday_	lag 0–14	Cumulative	RR	P_95_ vs. 0°C	Yes	Yes
Li et al., 2016 (23)	Poisson GLM + DLNM	TCN	TCN: Tmean__t_–Tmean__t–1_	lag 0–10	Cumulative/Single-day	RR	Per 1°C increase (ref = 0 °C)	Yes	No
Phakisi et al., 2025 (24)	Negative binomial mixed-effects + DLNM	DTR	T_max_ -T_min_	lag 0–7	Distributed lag	RR	P_95_ vs. 0°C	Yes	Yes
Qiu et al., 2015 (25)	Poisson GAM + DLM	DTR	T_max_–T_min_	lag 0–4	Cumulative	RR	Per 1 °C increase (ref = 0°C)	Yes	Yes
Wasilevich et al., 2012 (26)	Conditional logistic regression	TV	Max absolute temp change during exposure period	4–24 h	Single period	OR	Per 1 °F change (ref = time-stratified self-matched control periods)	Yes	Yes
Wei et al., 2020 (27)	GAM + stratified parametric	DTR	T_max_–T_min_	lag 0–4	Moving average (5-day)	RR	Per 1 °C increase (ref = 0 °C)	Yes	Yes
Wu et al., 2021 (28)	Conditional logistic regression	TV_0-7_	SD of T_max_ and T_min_ for current day + lag 0–7 days (16 values)	lag 0–7	Cumulative	RR	Per 1 °C increase (ref = 0 °C)	Yes	No
Xu et al., 2013 (29)	Poisson GLM + DLNM	DTR	T_max_–T_min_	lag 0–11	Cumulative	RR	Per 5°C increase above 10 °C (ref = P_50_)	Yes	Yes
Yan et al., 2021 (30)	Quasi-Poisson GLM + DLNM + linear threshold	TV_0-7_	SD of T_min_ & T_max_ for current+lag0-7 days (16 values)	lag 0–7	Cumulative	RR	Per 1 °C increase above min	Yes	Yes
Yu et al., 2024 (31)	Poisson GLM + DLNM	DTR	T_max_–T_min_	lag 0–14	Cumulative	RR	DTR-specific RRs vs. reference point	Yes	Yes
Zheng et al., 2023 (12)	DLNM + bivariate response surface model	DTR, TCN, TV_0−t_	DTR: T_max_–T_min_ TCN: T_mean_t_ − Tmean_t−1_ TV_0−t_: SD of daily T_min_ and T_max_ over the exposure window	DTR/TCN: 0–14 days; TV_0−t_: 0–7 days (TV_0−1_ to TV_0−7_)	Single-day lag+moving average/cumulative	RR	DTR: per P_95_ vs. Ref, TCN: P_5_ vs. Ref (low TCN / temperature reduction) TV_0−t_: per 1 °C increase	No	Yes

DTR, diurnal temperature range; T_max_, daily maximum temperature; T_min_, daily minimum temperature; TCN, temperature change between neighboring days; TV, temperature variability; TV_0−7_, temperature variability over lag 0–7 days; SD, standard deviation; GLM, generalized linear model; DLNM, distributed lag non-linear model; DLM, distributed lag model; GAM, generalized additive model; RR, relative risk; OR, odds ratio; Met. Adj., meteorological adjustment; Pol. Adj., pollutant adjustment.

The exclusion criteria were as follows: ① Articles with ineligible study designs, including reviews, commentaries, case reports, case series, cohort studies, cross-sectional studies, and ecological correlation studies. ② Basic experimental studies using animals or cell-based samples. ③ Studies without extractable effect sizes and 95% CI. Studies for which missing data could not be obtained after contacting the corresponding authors. ④ Duplicate publications. ⑤ Gray literature without formal peer review, such as conference abstracts, dissertations, and research reports. ⑥ Publications with unavailable full text.

### 2.4 Literature screening and data extraction

Two researchers independently performed literature screening in two phases: title and abstract screening and full-text review. We excluded clearly irrelevant records during the initial screening and retained uncertain articles for further evaluation. Full texts were assessed against predefined criteria, and all exclusion reasons were recorded. Any disagreements were discussed first, and unresolved conflicts were resolved by a third researcher. A standardized form was used for data extraction, and the extracted information was divided into five categories. Basic publication information included the first author, publication year, study location, and study period.

Study design included study type, study population, total number of asthma visits, and outcome measures. Exposure characteristics included exposure indicators (DTR, TCN, TV_0−t_), lag periods, cumulative exposure windows, and the optimal lag period reported by the authors. Effect estimates included effect size types (RR, OR), effect sizes, and 95% CIs per unit change in the exposure, and stratified results by age, gender, and season. Methodological details included statistical models, adjustment for confounders, and specific confounding factors.

For studies reporting effect sizes across multiple lag periods, we prioritized data from the authors’ specified optimal lag periods. For studies with multiple age subgroups, we extracted separate data for preschool children aged 0–6 years and school-aged children and adolescents aged 7–18 years. For studies presenting results for cold and warm seasons, we extracted independent effect sizes for each season. We emailed the corresponding authors to verify or supplement missing or ambiguous data. Studies for which missing data could not be obtained were excluded.

### 2.5 Risk of bias assessment

No validated tool exists for evaluating environmental epidemiological time-series biases. We applied the modified ROBINS-I (16) adapted from Lam et al. (17) and Khosravipour et al. (18), adding lag structure and model diagnostic domains that were absent from the original tool. Assessment criteria for the two extra domains followed their published standards: lag domains assessed selective reporting of lag results, whereas model domains evaluated the adequacy of temporal adjustment. We assessed ten domains, each graded low/probably low/probably high/high risks. According to the official ROBINS-I guidance, overall risk was determined by the worst single domain score. Two independent raters achieved 85% agreement (Kappa = 0.78), and conflicts were resolved by discussion or a third arbitration.

### 2.6 Statistical analysis

All statistical analyses were conducted in R 4.2.1 using the meta (v8.0) and metafor (v4.8) packages, with a two-tailed significance threshold set at *p* < 0.05. Given that asthma healthcare visits were rare outcomes across all included studies, all effect sizes (OR, excess risk percentage [ER%]) were unified to RR: ER% was converted via RR = 1 + ER%/100, and case-crossover ORs were directly treated as RR. All RR values were log-transformed (lnRR), with corresponding standard errors (SE) calculated for inverse-variance pooling.

A standardized harmonization workflow was implemented before pooling to address inconsistent exposure units and contrast types across studies, with separate meta-analyses run for distinct DTR contrast groups to ensure within-subgroup comparability. ① All temperature metrics were converted to Celsius. Per 5 °C DTR increments were rescaled to per 1 °C by dividing the lnRR and SE by 5; the single study reporting °F effects was converted to °C (1 °*F* = 0.556 °C) with lnRR and SE rescaled accordingly.② Linear subgroup: Only standardized per 1 °C DTR increment were pooled, representing linear risk changes from each 1 °C rise in diurnal temperature fluctuations.③ Extreme percentile subgroup: Studies comparing P_95_/P_99_ high DTR against reference DTR (<_P_50) were pooled separately. Despite minor differences in percentile cutoffs, all studies shared the same contrast logic (extreme vs. mild temperature fluctuations). Sensitivity analysis excluding P_99_ studies showed consistent pooled results. A subgroup difference test quantified heterogeneity driven by disparate exposure contrast definitions.④ Heterogeneous lag windows, calculation formulas, and percentile thresholds precluded universal standardization for TCN and TV_0-t_; quantitative meta-analysis was therefore omitted, and only narrative descriptive analysis was performed.

For consistent lag selection, we extracted each study’s self-reported optimal cumulative lag window identified via distributed lag non-linear models (DLNM). This extraction rule was not prespecified in PROSPERO and may have overestimated the pooled effects. We intended to run a unified lag 0–7 sensitivity analysis, but no separate lag 0–7 data were reported in primary studies; leave-one-out analysis was conducted to observe fluctuations in pooled effect sizes after excluding single studies individually.

Heterogeneity was assessed using Cochran’s Q test and I^2^ statistics. Fixed-effects models were used for low heterogeneity (P_Q_>0.1, I^2^ < 50%); otherwise, random-effects models were applied. Leave-one-out sensitivity analysis was performed to observe changes in the pooled estimates after excluding individual studies, including those with non-standard original exposure increments. Publication bias (funnel plots, Egger’s test, and trim-and-fill adjustment) was only assessed for subgroups with ≥10 studies.

## 3 Results

### 3.1 Study selection

**Figure 1:**
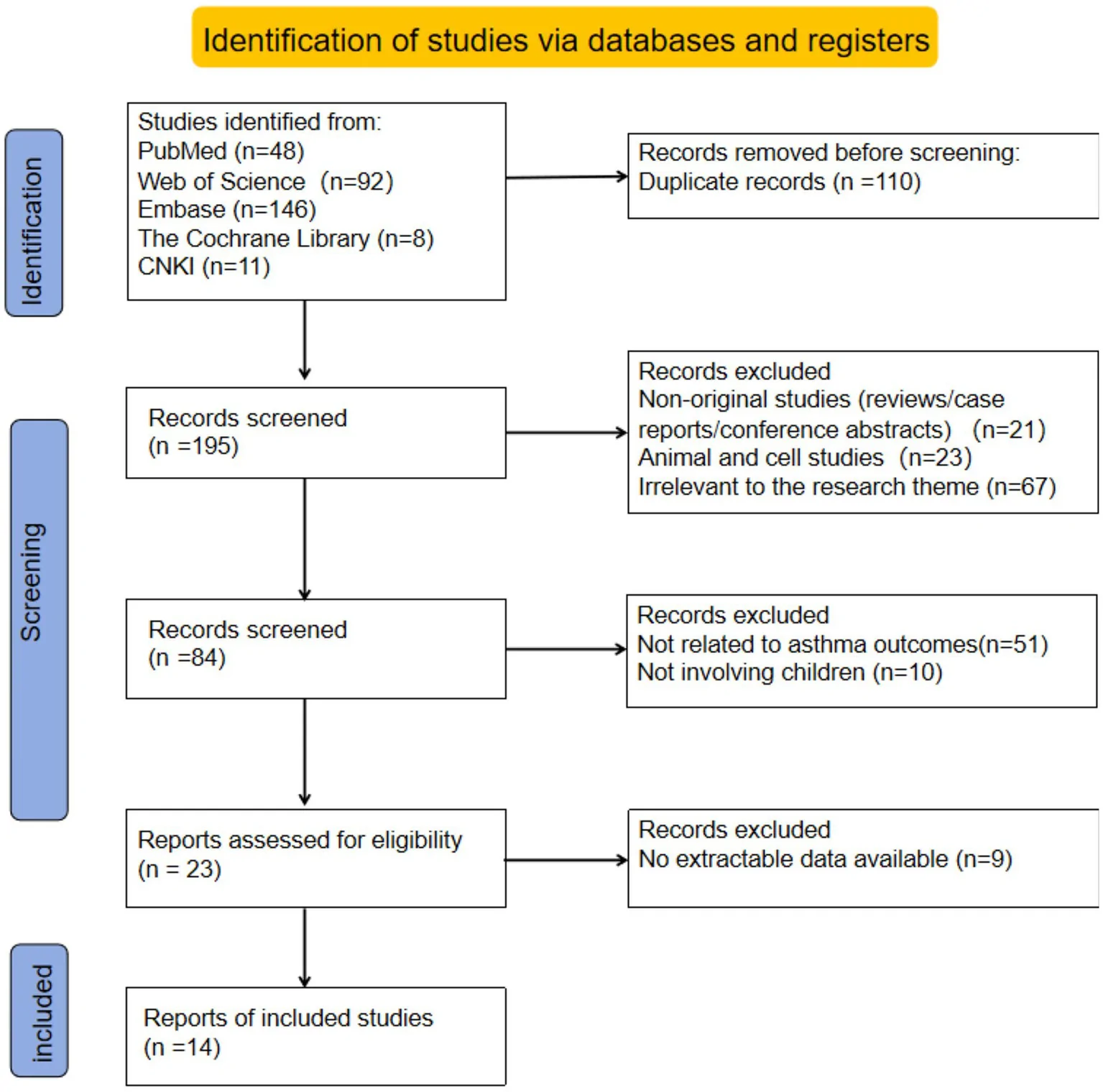
A flowchart of the systematic review process.

The literature search and screening followed the PRISMA 2020 guidelines (Figure 1). We retrieved 305 records from PubMed, Web of Science, Embase, Cochrane Library, and CNKI. A total of 110 duplicate records were removed. The remaining 195 articles were subjected to title and abstract screening. We excluded 111 irrelevant articles, including non-original studies and animal and cell-based experiments. This left 84 articles for full-text review. After full-text assessment, 61 articles were excluded. These studies did not focus on asthma-related visits or pediatric populations. Twenty-three studies remained for the final evaluation. Among them, nine were excluded due to unavailable quantitative effect sizes. Ultimately, 14 studies were included in this meta-analysis.

### 3.2 Characteristics of included studies

Fourteen eligible studies were included (12, 19–31). They covered five countries. The study period ranged from 2000 to 2020. The total number of asthma-related visits exceeded 4.5 million (Table 2). Twelve studies adopted an ecological time-series design (12, 19–25, 27, 29–31). The other two were stratified case-crossover studies (26, 28). Ten studies were conducted in China (12, 19–22, 25–27, 30, 31). The remaining four were from other countries (23, 24, 28, 29). All participants were children and adolescents aged 0–18 years. The outcomes included asthma outpatient visits, emergency visits, and hospitalizations. The exposure indicators included DTR, TCN, and TV_0-t_. The lag window ranged from 0 to 28 days. The majority of studies used distributed lag non-linear models (DLNM) to estimate cumulative effects. All studies were adjusted for basic confounders, such as temporal trends. Ten studies further controlled for meteorological factors and air pollutants (19, 20, 22, 24–27, 29–31) (Table 1).

**Table 2:** Baseline characteristics of included studies.

Study (author, year)	Country	Study period	Study design	Study population	Sample size	Outcome
Hu et al., 2020 (19)	Shanghai, China	2008.1.1–2017.12.31	Ecological time-series	Children and adolescents aged 0–17 years	23,103 emergency visits	Asthma emergency department (ED) visits
Hu et al., 2022 (20)	Shanghai, China	2008.1.1–2017.12.31	Ecological time-series	Children and adolescents aged 0–18 years	1,289,896 outpatient visits	Asthma outpatient visits
Wang et al., 2018 (21)	Shijiazhuang, Hebei, China	2012–2015	Ecological time-series	Children and adolescents aged 0–14 years	38,429 visits	Asthma outpatient visits
Lei et al., 2020 (22)	Shanghai, China	2008.1.1–2017.12.31	Ecological time-series	Children and adolescents aged 0–18 years	279,477 visits	Asthma outpatient visits
Li et al., 2016 (23)	Hefei, Anhui, China	2010.6–2013.7	Ecological time-series	Children and adolescents aged 0–14 years	17,022 visits	Asthma hospital admissions
Phakisi et al., 2025 (24)	Cape Town, South Africa	2009, 2014, 2019	Ecological time-series	Children and adolescents aged 6–13 years	7,753 visits	Asthma hospital admissions/outpatient visits
Qiu et al., 2015 (25)	Hong Kong, China	2004–2011	Ecological time-series	Children and adolescents aged 0–14 years	45,896 visits	Asthma ED visits
Wasilevich et al., 2012 (26)	Detroit, Michigan, USA	2000–2001	Time-stratified case-crossover	Children and adolescents aged 3–18 years	4,804 visits (descriptive); 4,871 cases + 16,546 controls	Asthma ED visits
Wei et al., 2020 (27)	Hefei, Anhui, China	2014.1.1–2015.12.31	Ecological time-series	Children and adolescents aged 0–18 years	19,064 hospital admissions	Asthma hospital admissions
Wu et al., 2021 (28)	Brazil	2000.1.1–2015.12.31	Time-stratified case-crossover	Children and adolescents aged 0–18 years	2,818,911 hospital admissions	Asthma hospital admissions
Xu et al., 2013 (29)	Australia	2003.1.1–2009.12.31	Ecological time-series	Children and adolescents aged 0–14 years	13,324 emergency visits	Asthma ED visits
Yan et al., 2021 (30)	Hefei, Anhui, China	2013.1–2016.12	Ecological time-series	Children and adolescents aged 0–18 years	12,994 visits (warm season); 13,361 visits (cold season)	Asthma hospital admissions
Yu et al., 2024 (31)	Lanzhou, Gansu, China	2013.1–2020.12	Ecological time-series	Children and adolescents aged 0–14 years	2,087 hospital admissions	Asthma hospital admissions
Zheng et al., 2023 (12)	Lanzhou, Gansu, China	2014.1–2019.12	Ecological time-series	Children and adolescents aged 0–18 years	23,678 outpatient visits	Asthma outpatient visits

### 3.3 Quality assessment of included studies

We assessed the risk of bias using the modified ROBINS-I tool (Table 3). The majority of the studies probably had a low overall risk of bias. Low risk of bias was observed in participant selection, exposure classification, outcome measurement, and conflicts of interest. Key potential biases existed in several domains. Four studies showed inadequate confounding adjustments (12, 21, 23, 28). One study had concerns regarding exposure windows and lag structures (21). One study reported deviations from the estimated exposure levels (21). Three studies had risks of selective outcome reporting (20, 21, 23). Overall, two studies were rated as low risk of bias, seven as probably low risk, and five as probably high risk. The included studies were of moderate quality. These potential biases should be considered when interpreting the results.

**Table 3:** Summary results of the risk of bias assessment in the included studies.

Study (author, year)	Criteria										
	D1	D2	D3	D4	D5	D6	D7	D8	D9	D10	Overall
Hu et al., 2020 (19)	L	L	L	PL	PL	PL	L	PL	L	L	PL
Hu et al., 2022 (20)	PL	L	PL	PL	L	L	PL	PH	L	PL	PH
Wang et al., 2018 (21)	H	PL	PL	PH	PH	L	PL	PH	L	PL	PH
Lei et al., 2020 (22)	PL	L	PL	PL	PL	L	PL	PL	PL	PL	PL
Li et al., 2016 (23)	H	PL	PL	L	PL	L	PL	PH	L	PL	PH
Phakisi et al., 2025 (24)	PL	PL	PL	L	PL	PL	PL	PL	L	PL	PL
Qiu et al., 2015 (25)	L	L	PL	L	L	L	L	PL	L	L	L
Wasilevich et al., 2012 (26)	L	PL	PL	L	L	L	PL	PL	L	PL	L
Wei et al., 2020 (27)	PL	PL	PL	PL	PL	L	PL	PL	L	PL	PL
Wu et al., 2021 (28)	H	PL	PL	L	L	L	PL	L	L	PL	PH
Xu et al., 2013 (29)	PL	PL	PL	L	PL	PL	PL	PL	PL	L	PL
Yan et al., 2021 (30)	PL	PL	PL	L	L	PL	PL	PL	L	L	PL
Yu et al., 2024 (31)	PL	PL	PL	L	PL	PL	PL	PL	PL	L	PL
Zheng et al., 2023 (12)	H	PL	PL	L	PL	PL	PL	PL	PL	L	PH

Green = low/probably‑low risk, orange = probably‑high risk, red = high risk; L, low risk; PL, probably low risk; PH, probably high risk; H, high‑risk; COI, conflict of interest.

D1: confounding, D2: selection of participants into the study, D3: classification of exposures, D4: exposure window and lag structure, D5: deviations from intended exposures, D6: missing data, D7: measurement of outcomes, D8: selection of the reported result, D9: conflict of interest, D10: other sources of bias.

### 3.4 Meta-analysis results

#### 3.4.1 Cumulative lag effects of DTR on asthma visits and hospitalizations

**Figure 2:**
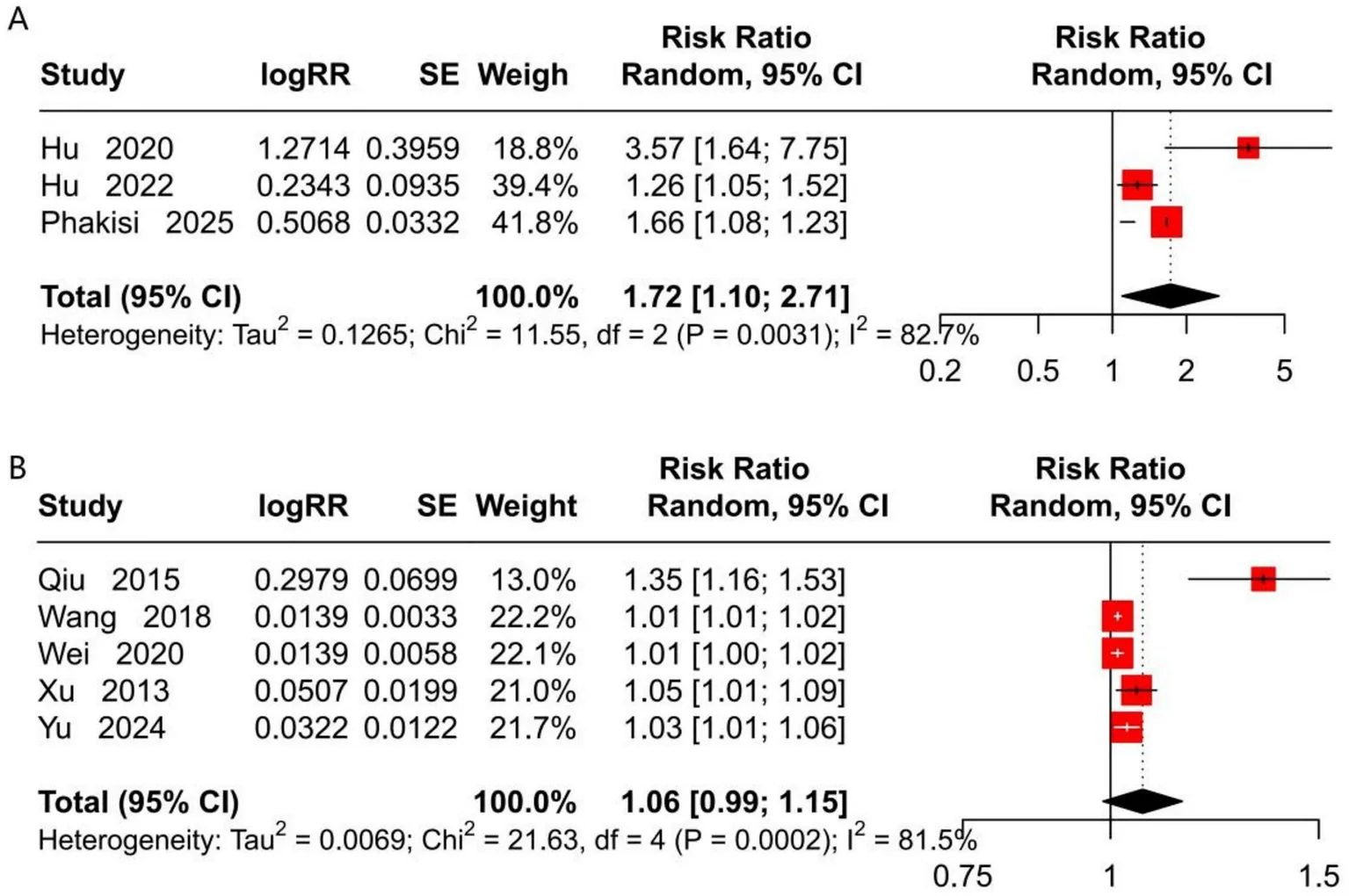
Forest plots of random-effects meta-analyses for associations between DTR and childhood asthma-related healthcare visits stratified by DTR exposure definition. Effect sizes are reported as RR with 95% CI. **(A)** Pooled results for studies defining DTR as extreme percentile temperature contrasts; **(B)** Pooled results for studies using a linear per 1 °C increment in the DTR metric.

We pooled cumulative DTR lag effects with lag windows spanning 0–28 days. We split the analyses into two subgroups due to inconsistent exposure definitions: per 1 °C linear DTR and extreme P_95_/P_99_ DTR. Subgroup interaction tests were used to compare the effect sizes. The pooled RR for the extreme-DTR subgroup (3 studies, Figure 2A) was 1.72 (95% CI: 1.10–2.71, *p* < 0.05), offering suggestive evidence for elevated childhood asthma-related visit risk linked to extreme DTR. Moderate to high heterogeneity was observed (I^2^ = 82.7%, *p* = 0.003). For the subgroup of per 1 °C DTR change (5 studies, Figure 2B), the pooled RR was 1.06 (95% CI: 0.99–1.15) without statistical significance, alongside high between-study heterogeneity (I^2^ = 81.5%, *p* < 0.001). This result requires cautious interpretation because of its high heterogeneity. Subgroup difference tests revealed significant disparities in effect sizes between the two exposure-definition groups under the random-effects model (*p* = 0.04), showing inconsistent exposure measurement as a major source of heterogeneity.

**Figure 3:**
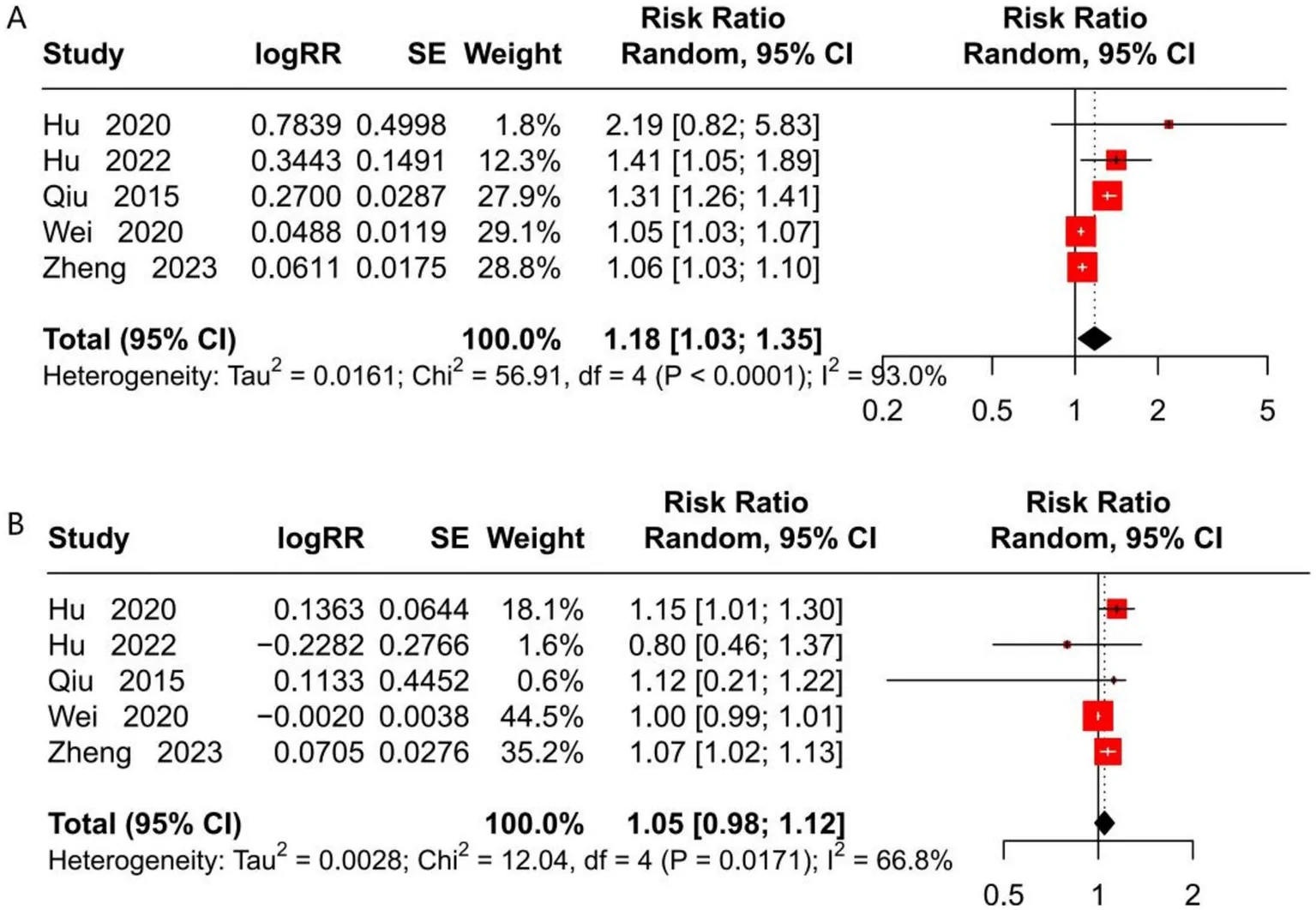
Forest plots of random-effects meta-analysis for the association between DTR and childhood asthma-related healthcare visits stratified by season. Effect sizes are presented as RR with 95% CI. **(A)** Pooled estimates for the cold-season subgroup; **(B)** Pooled estimates for the warm-season subgroup.

We further conducted a seasonal subgroup analysis (Figure 3). Five studies were included in the cold-season subgroup. Pooled results from the random-effects model showed that higher DTR was significantly associated with increased asthma-related visit risk (RR = 1.18, 95% CI: 1.03–1.35, I^2^ = 93%, *p* < 0.001). The cold-season result has limited generalizability owing to its extreme heterogeneity. The pooled RR was 1.05 (95% CI: 0.98–1.12) for the warm-season subgroup. However, this difference was not statistically significant. Subgroup difference tests revealed no significant difference between the two groups (*p* = 0.13). We did not perform an overall pooling due to varied seasonal definitions and climatic conditions across studies.

**Figure 4:**
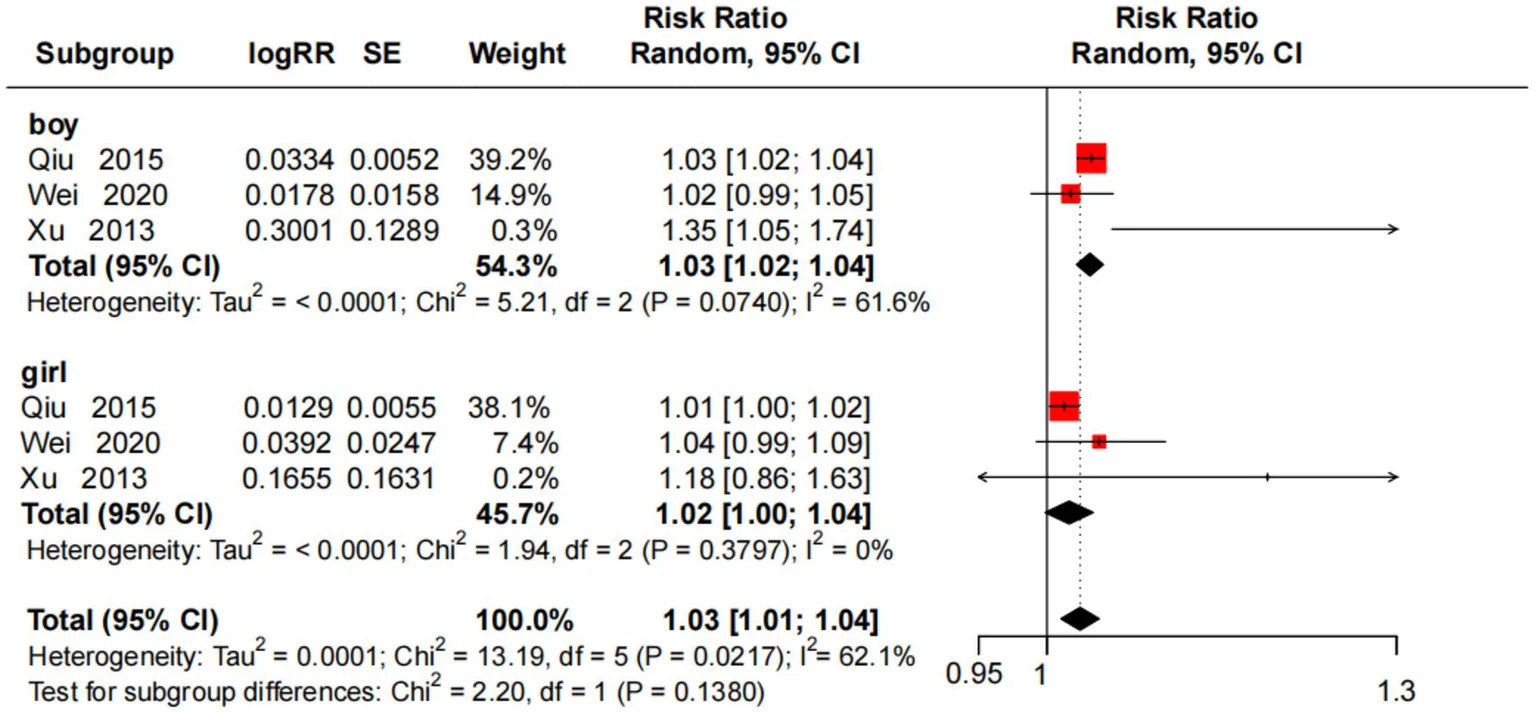
Forest plot of the random-effects subgroup meta-analysis evaluating associations between DTR and childhood asthma-related healthcare visits stratified by sex. Effect sizes are reported as RR with a corresponding 95% CI. A test for subgroup differences was performed to compare pooled effect magnitudes between boys and girls.

We also stratified the data by sex (Figure 4). For boys (3 studies), the pooled RR from the random-effects model was 1.03 (95% CI: 1.02–1.04, I^2^ = 61.6%). A higher DTR was significantly associated with an increased asthma-related visit risk in boys. The pooled RR for girls (3 studies) was 1.02 (95% CI: 1.00–1.04). This association reached marginal significance, with no between-study heterogeneity (I^2^ = 0%). The interaction test showed no statistically significant sex-based differences in the association between DTR and childhood asthma visits (*p* = 0.14).

**Figure 5:**
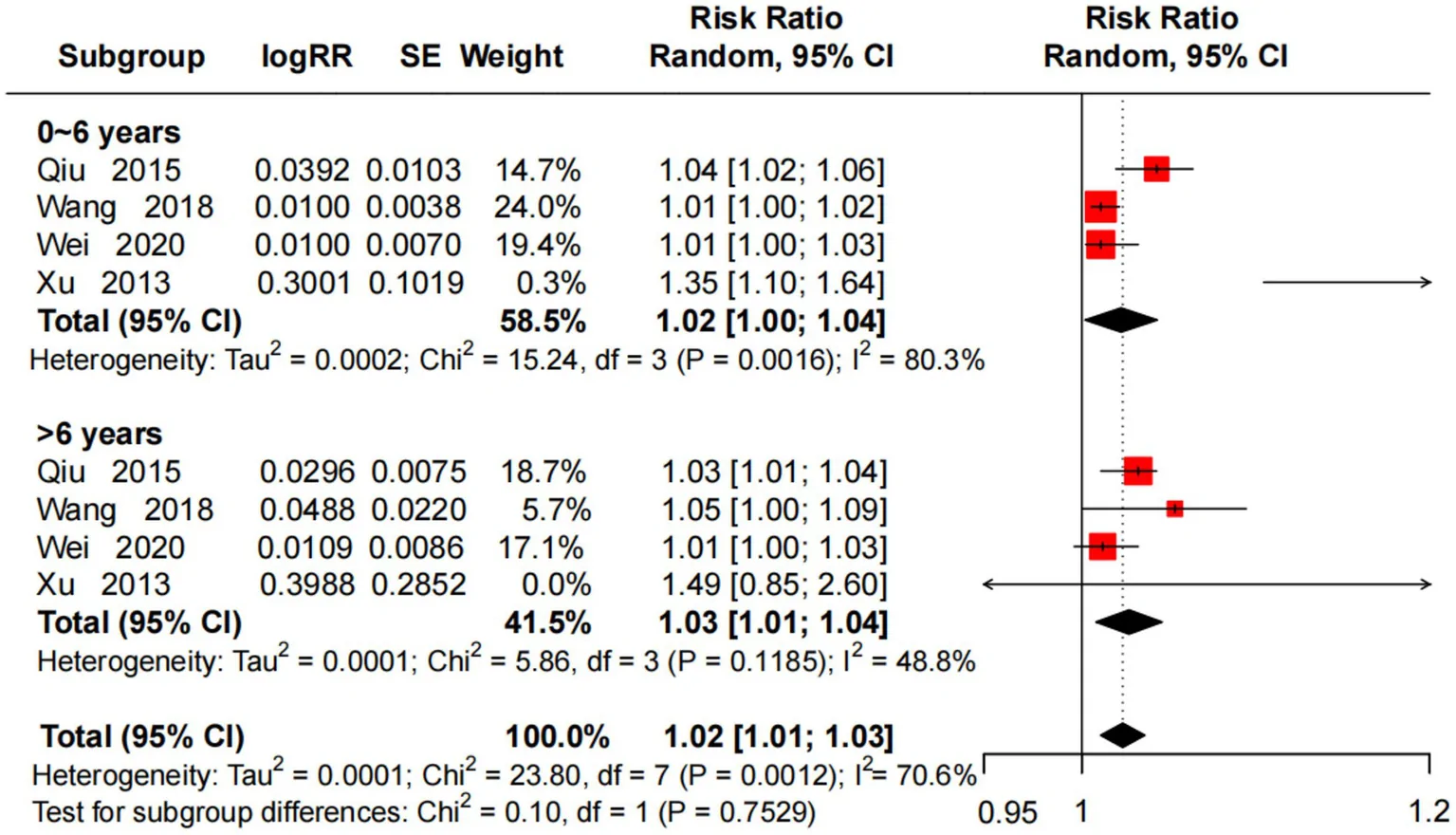
Forest plot of random-effects subgroup meta-analysis evaluating associations between DTR and childhood asthma-related healthcare visits stratified by age subgroups. Effect sizes are reported as RR with 95% CI. Subgroup difference testing was used to compare the pooled effect estimates between children aged 0–6 years and those who are aged 7–18 years.

Age-based subgroup analysis showed a significant increase in risk in both age groups (Figure 5). For preschool children aged 0–6 years (4 studies), the pooled RR under the random-effects model was 1.02 (95% CI: 1.00–1.04, I^2^ = 80.3%). High heterogeneity means that we cannot draw definitive conclusions for preschoolers. An increase of one unit in DTR corresponded to a 2% increase in asthma-related visit risk. For school-aged children aged 7–18 years (4 studies), the pooled RR was 1.03 (95% CI: 1.01–1.04, I^2^ = 48.8%). This group also showed a significant increase in risk. Subgroup difference tests revealed no significant difference between the two groups (*p* = 0.75).

#### 3.4.2 Associations of TCN and TV_0−t_ with childhood asthma-related visit risk

The number of studies on TCN and TV_0−t_ was limited. These studies also differed significantly in their exposure definitions, lag structures, and effect sizes. We did not perform a meta-analysis and instead adopted a descriptive analysis (Table 4). Among the four TCN studies, three identified elevated risks of asthma, while one showed non-significant associations. Seasonal discrepancies existed: Hu et al. (20) observed a cold-season RR of 0.776 (a protective trend), and warm-season TCN associations were mixed—Hu et al. (20) reported higher risk, whereas Zheng et al. (12) found protective effects. Two TV_0−t_ studies also yielded conflicting results: Wu et al. (28) and Zheng et al. (12) detected positive associations in Brazil and the cold season in Lanzhou, respectively, while Wasilevich et al. (26) reported a weak inverse association in the United States. Such divergent effect directions are likely attributable to inconsistent TV calculation methods across the original research.

**Table 4:** Associations of TCN/TV with childhood asthma-related visit risk.

Study (author, year)	Exposure type	Lag period	Effect measure	RR (95% CI)	Conclusion
Hu et al., 2022 (20)	TCN	Whole year: lag 0–14; Cold season: lag 0–14; Warm season: lag 0–14	Comparison of extreme values at P99	Whole year: 2.140 (0.290, 15.777); Cold season: 0.776 (0.555, 1.084); Warm season: 2.964 (1.636, 5.373)	Extreme TCN increased risk in the warm season; no significant link in the cold season
Li et al., 2016 (23)	TCN	lag 0–10	Per 1 °C increment	Warm season: 1.042 (1.009, 1.076)	Higher TCN increased the risk of childhood asthma hospitalization.
Zheng et al., 2023 (12)	TCN	Warm season: lag 0–14; Cold season: lag 0–14	Cumulative RR	Warm season: protective effect; cold season: 1.058 (1.009, 1.109)	TCN increased risk in the cold season and exerted a protective trend in the warm season
Wasilevich et al., 2012 (26)	TV	24 h	Per 1 °C increment	0.992 (0.984, 0.999)	Short-term TV showed a weak negative correlation with emergency visit risk
Wu et al., 2021 (28)	TV_0−t_	lag 0–7	Per 1 °C increment	1.010 (1.007, 1.014)	Elevated TV_0-t_ increased asthma hospitalization risk in children
Zheng et al., 2023 (12)	TV_0-t_	TV_0−1_ (warm); TV_0−3_ (cold)	Per 1 °C increment	Warm season: 0.999 (0.969, 1.030); Cold season: 1.039 (1.001, 1.077)	TV_0-t_ increased the risk in the cold season; no significant association was observed in the warm season.

TCN, temperature change between neighboring days; TV, temperature variability; TV_0−t_, temperature variability over specific lag days; RR, relative risk; CI, confidence interval; P99, 99th percentile.

#### 3.4.3 Sensitivity analysis

We conducted leave-one-out sensitivity analysis for the extreme DTR and cold-season subgroups. Recalculated pooled estimates showed no marked shifts in association direction or statistical significance after removing any single study, which only reflects the limited fluctuation of the subgroup results. We ran a supplementary sensitivity analysis using a unified lag 0–7 cumulative window to assess the heterogeneous lag selection effects. However, all primary studies only reported their single optimal lag without separate lag 0–7 effect sizes; therefore, this lag-standardized meta-analysis could not be performed. We used leave-one-out testing as an alternative to examine the potential impact of varying lag windows on our main findings.

#### 3.4.4 Publication bias

Each subgroup contained fewer than five studies. The sample size did not fulfill the minimum requirement for publication bias assessment. We did not perform Egger’s test, funnel plot analysis, or trim-and-fill correction.

## 4 Discussion

This meta-analysis synthesized existing observational data and provided preliminary evidence of a potential link between short-term temperature variability and pediatric asthma healthcare visit risk. Extreme DTR was significantly associated with a higher risk. Only a numerical trend of higher DTR effect sizes was observed in cold seasons, with no statistically significant seasonal interaction. Data on TCN and TV_0-t_ were too limited for meta-analysis, although descriptive results hinted at seasonal variation. These findings offer practical references for pediatric asthma prevention amid ongoing climate change.

Previous studies on the health impacts of DTR have primarily focused on cardiovascular diseases and older adult populations. A 2025 global systematic review and meta-analysis reported that each 1 °C increase in DTR increased cardiovascular hospitalization by 1.5%, with RR values of 1.02 for acute myocardial infarction and 1.04 for heart failure, alongside a 4.5% rise in stroke admission among older adults (32). Another systematic review of temperature variability and disease burden, including 38 studies, found that DTR and short-term daily temperature variability exerted stronger cardiovascular effects during hot seasons, whereas adverse respiratory effects were more prominent during cold seasons. Each 1 °C DTR elevation increased respiratory mortality by 0.7% and morbidity by 1.0% (33). Our pooled RR of 1.72 for extreme DTR among children was numerically higher than adult respiratory risk estimates, which may imply greater pediatric susceptibility to temperature fluctuations. Such age-based differences may stem from immature thermoregulatory centers, higher body surface area per unit weight, faster respiratory rates, and distinct behavioral exposure patterns in children. Basu et al. (34) reported that infants are more susceptible to temperature changes due to inadequate thermoregulatory responses. A regional study in Guangzhou also found a greater susceptibility to temperature drops during pediatric respiratory infection outpatient visits, especially in infants under 1 year of age (35). We detected no significant age-based differences in DTR effects, possibly due to inconsistent age grouping criteria and complexities in pediatric asthma diagnosis; however, the population-level vulnerability of children warrants further research.

Although no statistically significant seasonal differences were observed in our meta-analysis, the numerically higher effect estimate in the cold season was consistent with the findings of previous systematic reviews on temperature variability and respiratory health (33). Notably, most prior evidence has focused on adults and older adults. Although we observed a numerical seasonal trend in children, this pattern could not be statistically validated. Pathophysiologically, cold temperature fluctuations may increase the risk of asthma through several pathways. Cold air stimulates airway sensory nerves and induces bronchoconstriction, excess mucus, and epithelial injury, with worse responses in children with baseline airway hyperresponsiveness (36). Mechanistically, transient receptor potential (TRP) ion channels, particularly Transient Receptor Potential Ankyrin 1 (TRPA1), are activated at temperatures below 17 °C. Expressed in airway epithelial cells and vagal sensory fibers, activated TRPA1 promotes thymic stromal lymphopoietin (TSLP) release, activates type 2 innate lymphoid cells (ILC2), and drives eosinophilic airway inflammation (37, 38). In cold seasons, large diurnal temperature fluctuations expose children to mild daytime temperatures and abrupt nighttime or early-morning temperature drops, sustaining persistent airway mucosal stress (39). During cold periods, indoor heating lowers humidity and dries respiratory tracts; cold environments also sustain influenza and syncytial viruses, which may interact with allergic responses to amplify the DTR-related risk of asthma (40). Warmer weather enables heat dissipation via vasodilation and sweating, and higher humidity eases temperature-triggered airway irritation, which could partly explain the lower risk estimates observed in the warm-season subgroups. These mechanistic interpretations remain speculative, as the majority of the included studies were conducted in temperate Chinese regions with inconsistent seasonal classification schemes, restricting generalizability to tropical or subtropical populations.

The uncertain effects of TCN and TV_0−t_ identified in this study reflect a longstanding methodological challenge in temperature-variability research. The current literature adopts diverse calculation methods for temperature variability indicators, including daily temperature ranges, inter-day differences, and multi-day standard deviations, to capture the distinct temporal scales of temperature fluctuations. DTR reflects direct airway stress from daily thermodynamic cycles, whereas TV_0−t_ represents weather-scale atmospheric instability linked to air mass alternation and frontal activity, which may indirectly alter the risk of asthma by modulating air pollutant concentrations, pollen dispersion, and pathogen transmission. Weidmann et al. (33) similarly highlighted this methodological fragmentation and called for standardized exposure frameworks. We declined to pool the TCN and TV_0−t_ data because of inconsistent exposure definitions. This conservative approach underscores the urgent need for unified measurement criteria. Future studies should reference percentile-based standardization of absolute temperature metrics to establish consistent definitions and grading standards for DTR, TCN, and TV, thus enabling cross-study and cross-regional effect comparisons.

No significant effect modification by sex and age was observed (*p* = 0.14 and *p* = 0.75, respectively). Only a mild numerical trend of elevated DTR-related risk was observed in boys. This trend aligns with the reported sex differences in temperature-linked allergic rhinitis (41). We could not confirm sex-specific physiological stress responses to temperature changes, given the non-significant subgroup difference. Such weak numerical divergence may stem from greater outdoor exposure and hormonal influences in prepubertal males. DTR risk magnitudes were comparable for preschool and school-aged children, matching a prior pediatric respiratory study (42). While this numerical consistency suggests that temperature variability harms children across all age brackets, the lack of statistical separation across age strata may result from inconsistent age categorization and variable asthma diagnostic criteria for young children.

Despite consistent risk trends across sex and age subgroups, marked between-study heterogeneity limits the certainty of all pooled DTR risk estimates. This variation arises from a combination of methodological and contextual factors commonly noted in prior global temperature-health meta-analyses (7, 18): inconsistent exposure quantification approaches (linear per 1 °C increments versus P_95_/P_99_ extreme percentile contrasts), variable cumulative lag windows ranging from 1 to 28 days, diverse regional climate backgrounds, heterogeneous asthma outcome types (outpatient visits, emergency attendance, hospital admissions), two distinct observational designs with inherent differences in confounding control capacity, uneven adjustment for air pollutants and meteorological covariates, and variable overall risk-of-bias ratings across the included studies. We mitigated the impact of heterogeneity through random-effects models, stratified subgroup analyses, and supplementary lag-standardized sensitivity tests, but residual between-study variation remains, and all pooled estimates should be interpreted conservatively.

Our findings have direct public health implications. The 2023 Lancet Countdown report warned that climate change-related health risks are worsening under the current 1.14 °C global warming, with temperature variability hazards substantially underestimated (43). Coupled model intercomparison project phase 5 (CMIP5) projections indicate shifting TCN trends under global warming. The winter TCN may decline across mid- to high-latitude regions, whereas the summer TCN may increase over Europe (33). On synoptic scales, weakened polar thermal gradients may reduce Northern Hemisphere temperature variability, and increases are projected in Central and Eastern Europe. CMIP6 simulations of long-term continental DTR show inconsistent outputs across models. The majority of the simulations reproduce the observed declining DTR trend over global land areas (44). Such spatiotemporal heterogeneity requires targeted climate adaptation strategies. The observed association between extreme DTR and increased pediatric asthma visits suggests that DTR may serve as a potential complementary indicator for temperature-related asthma early warning systems rather than a replacement for conventional average temperature monitoring. The numerically higher risk estimates in cold-season subgroups indicate that temperate and frigid regions may consider incorporating DTR monitoring into existing pediatric asthma prevention frameworks alongside cold weather alerts to help families and schools enhance preparedness and asthma management on days with large temperature fluctuations.

This study has several limitations. First, formal publication bias tests and certainty-of-evidence grading (e.g., Grading of Recommendations Assessment, Development, and Evaluation [GRADE]) were not feasible because all subgroups included fewer than 10 studies. Small subgroup sizes and a moderate overall risk of bias limit the strength of the current evidence. Second, substantial between-study heterogeneity was present in key pooled analyses, driven by inconsistent exposure metrics (per 1 °C increment vs. extreme percentiles), variable lag windows, and differing confounder adjustment strategies. Extracting study-specific optimal lag periods may have slightly inflated the pooled effect estimates. We planned to conduct a unified lag 0–7 sensitivity analysis to address this bias, but the primary studies only reported a single optimal lag without stratified lag data. Leave-one-out analysis was applied to check for changes in the pooled estimates. Third, the majority of the included studies were conducted in China, restricting global generalizability. For TCN and TV_0-t_, limited studies and inconsistent calculation methods precluded quantitative meta-analysis; therefore, no definitive conclusions can be drawn for these indicators. Fourth, ambient station-based exposure data cannot capture individual microenvironmental differences, potentially underestimating the true health effects. The majority of studies also did not adjust for biological aerosols (e.g., pollen and mold), leaving residual confounding factors (45, 46). Fifth, the lack of individual-level data on asthma severity and medication use prevents the identification of high-risk subgroups. Future multicenter studies with standardized exposure definitions and unified lag frameworks are needed to further validate the impact of temperature variability on pediatric asthma.

We performed a qualitative assessment of evidence certainty according to the PRISMA guidelines; a full GRADE evaluation was not conducted because of the limited number of studies per subgroup. The judgments covered four domains: risk of bias, between-study heterogeneity, effect estimate precision, and the ability to assess publication bias. Overall evidence certainty was rated low to moderate for the primary association between extreme DTR and increased childhood asthma visits and low for linear per 1 °C DTR effects and seasonal, sex, and age subgroup modifications. No definitive causal conclusions can be drawn from the current observational evidence.

## 5 Conclusion

Extreme DTR may be a potential risk factor for elevated pediatric asthma-related visit risk. Risk estimates were numerically larger in the cold-season subgroups, without statistically significant subgroup differences across seasons, sex, and age. Our observational findings only provide tentative clues; standardized multiregional studies are needed to clarify the independent effects of TCN and TV indicators and strengthen certainty of evidence.

## Data Availability

The original contributions presented in the study are included in the article/Supplementary material, further inquiries can be directed to the corresponding author.
