# Supplementary material for: Association between short-term ambient temperature variability (TV, DTR, and TCN) and asthma-related healthcare visit risk in children and adolescents: a systematic review and meta-analysis
Source: Front Public Health. 2026 Aug 13;14:1902965. doi: 10.3389/fpubh.2026.1902965 (PMC13518597; doi:10.3389/fpubh.2026.1902965)
Supplement: Supplementary file 1 [file Table_1.DOCX]

Supplementary Material

## 1.1 Supplementary Tables

Table S1 Full Database-Specific Search Strategies for the Systematic Review

| Database Name | Time Filter | Language Filter | Full Search Strategy |
| --- | --- | --- | --- |
| PubMed | From database inception to May 27, 2026 | English | ("Diurnal Temperature Range"[Title/Abstract] OR "DTR"[Title/Abstract] OR "Temperature Variability"[Title/Abstract] OR "Temperature Variation"[Title/Abstract] OR "TCN"[Title/Abstract] OR "Temperature change between neighboring days"[Title/Abstract] OR "Ambient temperature fluctuation*"[Title/Abstract]) AND ("Asthma"[Mesh] OR "Asthma"[Title/Abstract] OR "Wheeze*"[Title/Abstract]) |
| Web of Science Core Collection | From database inception to May 27, 2026 | English | (("Diurnal Temperature Range" OR DTR OR "Temperature Variability" OR "Temperature Variation" OR "Temperature Fluctuation*" OR TCN OR "Temperature change between neighboring days" OR "Inter-day temperature variation" OR "Ambient temperature change") AND (Asthma OR Wheez*)) |
| Embase | From database inception to May 27, 2026 | English | ('diurnal temperature range'/exp OR 'diurnal temperature range' OR DTR OR 'temperature variability' OR 'temperature variation' OR 'temperature fluctuation*' OR 'thermal fluctuation*' OR 'day-night temperature' OR 'temperature change' OR 'inter-day temperature variation' OR 'temperature change between neighboring days' OR TCN) AND ('asthma'/exp OR asthma OR wheeze*) |
| Cochrane Library | From database inception to May 27, 2026 | English | #1 (diurnal temperature range OR DTR OR "temperature variability" OR "temperature variation" OR "temperature fluctuation*" OR "thermal fluctuation*" OR "day-night temperature" OR "temperature change" OR "inter-day temperature variation" OR "temperature change between neighboring days" OR TCN)  #2 (Asthma OR Wheeze* OR "Respiratory Hypersensitivity" OR "Bronchial Hyperreactivity")  #3 #1 AND #2 |
| CNKI | From database inception to May 27, 2026 | Chinese | #1 昼夜温差 [主题词] OR 温度变异性 [主题词] OR 温度波动 [主题词] OR 气温变化 [标题 / 摘要] OR 气温波动 [标题 / 摘要] OR 昼夜温差 [标题 / 摘要]  #2 哮喘 [主题词] OR 喘息 [主题词] OR 支气管哮喘 [标题 / 摘要] OR 呼吸道疾病 [标题 / 摘要]  #3 #1 AND #2 |

Note：CNKI = China National Knowledge Infrastructure.

Table S2 Judgment criteria for custom Domain D4 (Exposure window and lag structure)

| Risk Grade | Grading standard for D4 Exposure window and lag structure |
| --- | --- |
| Low risk | Lag windows and lag structures were predefined; all lag-specific effect estimates fully reported without selective reporting. |
| Probably low risk | Primary lag results fully reported; only minor lag sensitivity analyses were omitted. |
| Probably high risk | Multiple lag windows were analyzed, yet only significant lag results were selectively reported. |
| High risk | Lag definitions were adjusted post-hoc after observing outcomes; only significant cumulative lag effects were presented. |

Note: Overall risk of bias for each study was determined by the highest risk grade across all ten domains (D1–D10), consistent with original ROBINS-I guidance.
